# Addressing Healthcare Waiting Time Challenges in Canada: Insights From Emerging Initiatives

**DOI:** 10.34172/ijhpm.8986

**Published:** 2025-09-22

**Authors:** Mohammad Hajizadeh, Faramarz Jalili

**Affiliations:** School of Health Administration, Faculty of Health, Dalhousie University, Halifax, NS, Canada.

**Keywords:** Waiting Times, Healthcare Access, Initiatives, Canada

## Abstract

Canada’s public healthcare system faces persistent challenges with waiting times. Prolonged delays lead to adverse physical and mental health outcomes, higher treatment costs, and economic burdens for patients and families. This editorial examines the drivers of extended wait times and policy responses at both provincial and federal levels. Contributing factors include systemic features of the Canadian healthcare system, such as shared federal–provincial jurisdiction, along with staffing shortages, population aging, structural inefficiencies, and poorly integrated health information technology. Provinces have introduced strategies such as digital health solutions, capacity expansion, workforce innovations (including Physician Assistants [PAs]), and expanded scopes of practice for pharmacists. At the federal level, a 10-year $196.1 billion investment announced in 2023 is supporting these initiatives. While such measures indicate progress, wait times remain a significant concern. Achieving equitable and timely access will require coordinated and sustained strategies that address systemic challenges and deliver long-term improvements.

## Prolonged Waiting Times in Healthcare and its Consequences in Canada

 Healthcare in Canada is provided through a publicly funded system known as Medicare, which often faces increased demand due to the absence of prices and limitations on the availability and supply of health services. As a result, for non-life-threatening conditions, prolonged waiting times for healthcare services are common, leading to patient dissatisfaction and potential deterioration in their health status. While similar to other Organization for Economic Co-operation and Development (OECD) countries, Canadian patients are largely shielded from direct health service costs through taxation or public insurance, the Canadian Medicare model is distinct in that it excludes universal coverage for prescription drugs, allied health services, and long-term care. Under the *Canada Health Act*, the federal government sets national standards through criteria such as universality, accessibility, comprehensiveness, portability, and public administration, while provincial governments hold primary responsibility for healthcare delivery.^1^ This shared jurisdiction often results in fragmented policies and uneven performance across provinces, contributing to systemic strain and exacerbating inequities in access. Unlike many other OECD systems, Canada does not allow a parallel private option for physician and hospital services. Consequently, all demand must be absorbed within the public system, increasing strain and contributing to longer patient wait times. A further challenge is the pronounced variation in wait times, both across provinces and among different socioeconomic groups,^1^ underscoring the need for equity-focused reforms.

 The issue of prolonged waiting times is a significant public health concern and a debated barrier to healthcare access for Canadian patients.^2,3^ A recent Canadian report^3^ highlights record-high wait times for healthcare services, with total waits—from general practitioner referral to elective specialist treatment across 12 specialties and 10 provinces—increasing from 25.6 weeks in 2021 to 27.4 weeks in 2023. The 2023 wait time represents a 195% increase from 1993, when it was just 9.3 weeks. Individuals who experience long waiting times to access healthcare services often face adverse physical and mental health outcomes.^4,5^ For instance, delayed treatment for patients with cancer or cardiovascular disease can increase the risk of morbidity and mortality.^4,6^ Longer waiting times are directly linked to lost opportunities for effective care, deteriorated health outcomes, higher treatment costs, an increased burden while living with a disability, and loss of income.^4-7^ Implementing efficient approaches to ensure timely access to healthcare services can help prevent serious health complications, improve mental health and quality of life, and reduce overall public healthcare cost.^3,8^

## Key Factors Contributing to Healthcare Waiting Times in Canada

 The first step in developing an effective strategy to reduce waiting time is to identify the multifaceted systemic and policy challenges, as well as the underlying barriers contributing to delays in accessing healthcare services for the Canadian population.^9^ In addition to broader structural features of the Canadian healthcare system (shared federal–provincial jurisdiction and the absence of a parallel private option, which create system-wide pressures that exacerbate wait time challenges), healthcare staffing shortages, including physicians, nurses, and specialists, remain one of the most significant factors causing delays in access. These shortages contribute to overworked and exhausted professionals, as well as the geographic maldistribution of healthcare providers.^9^ Such challenges compromise the quality of care, limit hospital capacity to admit patients, exacerbate overcrowding, and prolong wait times for essential services.^10^

 The increasing proportion of older adults in the Canadian population also plays a crucial role in prolonged waiting times.^11,12^ This demographic shift places substantial demand on the healthcare system, requiring more complex and specialized medical care. Consequently, there is increased pressure on healthcare resources, leading to longer wait times for services such as joint replacement surgeries and chronic disease management.^11,13^

 Like other publicly funded systems, Canada’s universal medical coverage contributes to wait times by creating a disconnect between service utilization and their associated costs. Since patients are not directly exposed to the full costs of healthcare services, this can lead to unnecessary treatments and contribute to gridlock in hospitals.^14^ Additionally, budget constraints and limited funding further strain the healthcare system’s capacity to meet demand. Insufficient resources hinder a hospital’s ability to expand services, hire additional or better-qualified staff, sustain training programs, and invest in critical infrastructure, technology, and monitoring systems.^15^

 Furthermore, poorly integrated health information technology and ineffective communication within many healthcare facilities contribute significantly to the current wait time challenges in Canada. Many organizations lack the technological infrastructure needed to streamline referral processes, prioritize patients, and reduce wait times.^16^ While several organizations have adopted internal electronic health records, these systems often lack interoperability with external systems across the healthcare sector. Thus, many facilities still rely on faxing as the primary mode of communication between providers, a method widely criticized for its increased risk of errors and miscommunication. Moreover, providers often need to redirect referrals if a specialist’s wait time is too long or if no response is received, wasting valuable time on re-ordering, analyzing, and faxing test results, only to restart the referral process. This inefficiency prolongs overall wait times for patients. The lack of effective connectivity in healthcare service facilities leads to administrative errors in processes, communication, and practices, ultimately increasing wait times for specialist consultation and reducing the capacity of primary care physicians to take on new patients.^16^

 Beyond these factors, epidemic outbreaks and diseases, such as the COVID-19 pandemic, have exacerbated these challenges, amplifying their impact and highlighting the severity of the systemic flaws contributing to prolonged wait times.^17^

## Insights From Emerging Key Initiatives to Reduce Wait Times in Canada

Table presents current initiatives for enhancing healthcare management in Canada, aimed at reducing wait times at both the federal level and across Canadian provinces.^18-20^ Key federal initiatives include a substantial $196.1 billion investment announced in 2023 to be distributed over ten years, supporting provincial and territorial healthcare improvements. Additionally, national strategies such as palliative care programs and wait time guarantees play a crucial role in enhancing patient access to timely and quality care.

**Table:** Current Initiatives for Enhancing Healthcare Management in Canada to Reduce Wait Times

**Level**	**Key Initiatives**	**Overview**
**Federal**		
	Healthcare Funding Agreements	The federal government announced a $196.1 billion investment in 2023 over 10 years to support provincial and territorial healthcare improvements, with funding agreements in place
	National Strategy for Palliative Care	The federal government has expanded palliative care services across Canada to meet the needs of an aging population, with the strategy being implemented
	Wait Time Guarantees	The province has implemented guarantees for procedures like hip replacements to ensure timely access, with policies in place. Wait time guarantees are not federally mandated but are supported by federal funding and guidance
	Digital Health Initiatives	The government has promoted the use of electronic health records and digital health solutions to improve service efficiency, with initiatives ongoing
**Provincial**		
British Columbia	Surgical Renewal Plan	Increased operating room hours Expanded surgical capacity Utilized private surgical clinics Adding Saturdays and Sundays to the schedule
	Activity-Based Funding	Allocates funds based on the actual services provided, incentivizing hospitals to increase efficiency and reduce wait times
	PAs	PAs are recognized healthcare professionals who work under the supervision of physicians and provide medical care as part of a team
Alberta	Patient Flow Platform	Monitor patient stays, assess discharge readiness, and support optimal care to enhance patient flow and reduce wait times
	PAs	PAs are recognized healthcare professionals who work under the supervision of physicians and provide medical care as part of a team
	Pharmacists’ Expansion of Scope	Pharmacists can prescribe medications, monitor chronic diseases, interpret lab results, assess small injuries on the spot, and deliver every recommended vaccine Alberta has also introduced pharmacist-led clinics, which improve primary care access and reduce unnecessary emergency room visits
Saskatchewan	Saskatchewan Surgical Initiative	Reduce surgical wait times by increasing surgical capacity, optimizing operating room efficiencies, and implementing pooled referrals to ensure timely access to care Supported by centralized wait-list registry and standard prioritization across the province, pooled referrals *via* an online Specialist Directory, and increased capacity through publicly funded private clinics Enhanced by expanded perioperative nursing resources and a cultural shift toward patient-centered and collaborative governance
	Virtual Care Services	Telehealth options for rural and remote communities
	PAs	PAs are recognized healthcare professionals who work under the supervision of physicians and provide medical care as part of a team
Manitoba	Diagnostic Imaging Expansion	Increased the availability of MRI and CT scans to reduce wait times for diagnostics, with new equipment installed
	PAs	PAs are recognized healthcare professionals who work under the supervision of physicians and provide medical care as part of a team
Ontario	Digital and Virtual Care Initiatives	Reduce in-person wait times Improve accessibility
	Hospital Expansion Projects	Increase healthcare capacity Reduce waiting time
	PAs	PAs are recognized healthcare professionals who work under the supervision of physicians and provide medical care as part of a team
Quebec	New Quebec Health Plan “Human and Efficient — Plan to implement necessary health changes”	Focus on accelerating access to front-line health services through a one-stop phone service Reducing emergency room wait times Improving working conditions for nurses More home-care services for seniors
Nova Scotia	Pharmacists’ Expansion of Scope	Allowed pharmacists to prescribe for minor ailments and manage chronic diseases, with the policy in effect
	Patient Navigators	Introduced navigators to help patients access services quickly and efficiently, with the program operational
	Collaborative Emergency Centers	Integrated paramedics, nurses, and doctors to provide 24/7 care in rural areas, with centers operational
	Single-Entry Models	Introduced centralized intake systems for specialist referrals to streamline care, with systems in place
	PAs	PAs are recognized healthcare professionals who work under the supervision of physicians and provide medical care as part of a team
Prince Edward Island	Mental Health Mobile Crisis Units	Deployed mobile units to provide crisis intervention and reduce ER visits, with units operational
	Cataract Surgery Expansion	Opening of new cataract outpatient clinic cutting into lengthy wait times for Prince Edward Islanders with blurred vision
	PAs	PAs are recognized healthcare professionals who work under the supervision of physicians and provide medical care as part of a team
Newfoundland and Labrador	Telehealth Services	Expanded telehealth for rural communities to improve healthcare access, with services available
	Mental Health and Addictions Strategy	This plan has four main pillars that form the basis of the 54 recommendations: promotion, prevention, and early intervention; focusing on the person; improving service access, collaboration, and continuity of care; including all people everywhere
New Brunswick	Stabilizing Health Care: An Urgent Call to Action	This plan sets five very specific action areas and timelines for improvements
	PAs	PAs are recognized healthcare professionals who work under the supervision of physicians and provide medical care as part of a team

Abbreviations: ER, emergency room; PAs, Physician Assistants; MRI, magnetic resonance imaging; CT, computed tomography. *Note:* Initiatives to reduce wait times in the three territories viz., Yukon, Northwest Territories, and Nunavut are not summarized in the table due to limited initiatives and information.

 Canadian provinces share many initiatives aimed at reducing wait times; however, their approaches and programs vary significantly. Each province tailors its approaches to address unique needs, challenges, disease patterns, demographics, facilities, and socioeconomic conditions. As outlined in Table, these initiatives span diverse directions, with several common themes emerging:

- *Digital Health Initiatives*: Several provinces, including Ontario and Saskatchewan, have implemented virtual care services to reduce in-person wait times and improve accessibility. Ontario has been at the forefront of digital care initiatives, while Saskatchewan has prioritized expanding these services to rural and remote communities.
- *Capacity Expansion*: Several provinces have launched substantial expansion projects. Ontario has initiated hospital expansion projects, while British Columbia has introduced a Surgical Renewal Plan, emphasizing extended operating room hours and increased surgical capacity. Manitoba has concentrated on diagnostic imaging expansion, with a particular focus on expanding magnetic resonance imaging (MRI) and computed tomography (CT) scan availability.
- *Workforce Innovation*: A notable initiative in workforce innovation is the widespread adoption of Physician Assistants (PAs) across multiple provinces, including Ontario, British Columbia, Alberta, Saskatchewan, Manitoba, Nova Scotia, Prince Edward Island, and New Brunswick. Working under physician supervision, PAs play a vital role in healthcare teams, enhancing service delivery capacity and improving access to care.
- *Specialized Programs*: Various provinces have implemented targeted initiatives to address specific healthcare needs. Nova Scotia has expanded pharmacists’ practice scope and introduced patient navigators, while Prince Edward Island has focused on mental health mobile crisis units and cataract surgery expansion.

Some provinces have reported positive outcomes from these emerging initiatives.^11,12^ As an example, by introducing clinical assistants to handle non-specialist tasks, surgeons were able to double their capacity for hip and knee replacements, reducing wait times from 12-18 months to 6-12 months in Winnipeg’s Concordia Hospital.^19^ The adoption of digital platforms for online appointment scheduling has been instrumental in optimizing time slots and reducing patient wait times. These systems facilitate efficient management of appointments, minimizing delays and improving patient care. The Saskatchewan Surgical Initiative is another successful example of policies introduced to reduce waiting times. By March 31, 2014, the number of patients waiting longer than three months for surgery had dropped to 3824, a 75% reduction compared to the 15 352 patients waiting over three months at the start of the initiative in 2010. Importantly, this initiative preserved universal access despite private involvement and showed that combining targets, centralized systems, and capacity expansion can significantly reduce surgical wait times.^20^ Workforce innovation through the integration of PAs has demonstrated measurable impact on reducing wait times in Canada. For example, in the Winnipeg Regional Health Authority’s Concordia Hospital, the adoption of a double-operating-room model supported by PAs led to a 42% increase in hip and knee replacement and a reduction in median wait times from 44 weeks to 30 weeks compared to the preceding year.^19^ While the available evidence highlights improved patient access and reduced wait times from the implementation of these initiatives, a comprehensive understanding of their impact remains limited due to the recent implementation of most initiatives. Therefore, further evaluation and long-term monitoring are needed to assess their overall effectiveness.

 Despite ongoing efforts, significant challenges persist in the healthcare system, including workforce shortages and limited funding, which contribute to prolonged wait times for healthcare in Canada. Addressing these issues requires a comprehensive, collaborative approach involving federal, provincial, and territorial governments. Effective strategies to address limited funding concerns must include investments in data analytics to optimize resource allocation, patient flow, and demand forecasting while supporting evidence-based decision-making. These improvements enhance efficiency, enabling healthcare systems to make the most of available resources. In parallel, tackling workforce shortages through targeted recruitment and retention of healthcare professionals, combined with innovative care models such as team-based care and virtual clinics, can significantly improve accessibility and operational capacity.

 Beyond overall wait times, equity remains a pressing concern. Canadians with lower incomes consistently experience longer waits than higher-income groups, despite universal insurance. Provincial variation further compounds the issue, as differences in demographics, policies, and service delivery create significant inequities in access.^1^ Reforms must therefore aim not only to reduce average wait times but also to enhance fairness in access. One proposed solution is the introduction of a parallel private system, intended to ease pressure on the public sector by reducing demand and enabling more timely access to care. International experience, such as in the United Kingdom and Australia, suggests that mixed public–private models can expand capacity. Yet such arrangements also carry risks, including the diversion of health professionals from the public system and the potential for widening inequities, with wealthier patients gaining faster access.

 It is important to recognize that the drivers of wait times vary by service, and so do the solutions. For example, delays in diagnostic imaging can be reduced by applying appropriateness criteria, specialist visits can be streamlined through single-entry referral models, and surgical wait times can be improved with team-based models of care. These cases highlight the need for targeted, service-specific strategies to reduce delays across the healthcare system. Regardless of the strategy adopted, reforms must place patient needs and preferences at the center to enhance satisfaction and achieve better health outcomes. Addressing the interrelated challenges driving wait times through a coordinated and sustained approach will be essential for reducing delays and ensuring equitable access to healthcare for all Canadians.

## Conclusions

 Prolonged wait times remain a significant challenge in Canada’s publicly funded healthcare system. Contributing factors include systemic features of Canada’s healthcare system, workforce shortages, population aging, outdated health information technology, and structural inefficiencies. Variations in resource allocation, infrastructure, and demographics across provinces further exacerbate differences in access to healthcare. While emerging initiatives at the federal and provincial levels represent progress, wait times continue to be a major challenge in Canada. Ensuring timely care for all Canadians and addressing inequities in healthcare access will require coordinated national strategies to foster long-term sustainability. Collaboration between federal and provincial governments, coupled with the implementation of evidence-based initiatives, is essential to achieve efficient, equitable, and timely healthcare access for all Canadians. Canada’s wait time challenges require not only policy innovation but also stronger evidence. Accordingly, there is a need for more rigorous evaluations of emerging initiatives, particularly long-term studies that could assess their sustainability, scalability, and impact on both efficiency and equity of access.

## Ethical issues

 Not applicable.

## Conflicts of interest

 Authors declare that they have no conflicts of interest.
